# Combined association of the triglyceride glucose index and body roundness index with cardiovascular disease in middle-aged and elderly persons with diabetes: a CHARLS-based cohort study

**DOI:** 10.3389/fnut.2025.1724178

**Published:** 2026-01-14

**Authors:** Xinbiao Fan, Yongchun Liang, Jun Ge, Xitong Sun, Xiaofei Geng, Boyu Zhu, Yuxin Kang, Zheng Zhang, Junping Zhang

**Affiliations:** 1 First Teaching Hospital of Tianjin University of Traditional Chinese Medicine, Tianjin, China; 2 National Clinical Research Center for Chinese Medicine, Tianjin, China; 3 Affiliated Hospital of Jiangxi University of Traditional Chinese Medicine, Nanchang, Jiangxi, China

**Keywords:** body roundness index, cardiovascular disease, CHARLS, diabetes, triglyceride glucose index

## Abstract

**Background:**

Insulin resistance and visceral obesity are key pathologic mechanisms of CVD. However, the combined effect of the triglyceride glucose (TyG) index and body roundness index (BRI) on CVD risk in the diabetic population has not been thoroughly investigated.

**Methods:**

The cohort study used data from four waves of the China Health and Retirement Longitudinal Study (CHARLS) conducted from 2011 to 2018, involving 1,010 participants with diabetes. Participants were categorized according to the median TyG index and/or BRI. Cox proportional risk regression models were used to examine the individual and joint associations of the two metrics with CVD risk. The study further estimated additive and multiplicative interaction effects.

**Results:**

During a median follow-up of 7 years, 251 participants developed CVD. The study confirmed a significant joint association between TyG index and BRI and the development of CVD in middle-aged and elderly persons with diabetes. Specifically, after adjusting for confounders, participants with both high TyG index and high BRI had a 123% increased risk of CVD compared with participants with both low TyG index and low BRI, and 85% for high BRI alone. In addition, the study did not find an additive and multiplicative interaction between BRI and TyG index on CVD.

**Conclusion:**

This study found that high TyG index and high BRI were significantly associated with increased risk of new-onset CVD in a Chinese middle-aged and elderly diabetic population, and the combined assessment of the TyG index and BRI enhanced the prediction of CVD.

## Graphical abstract

**Figure:**
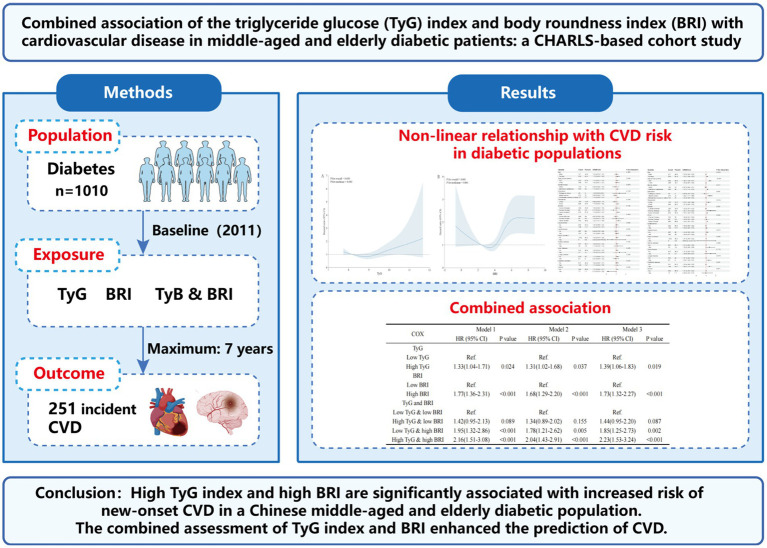

## 1 Introduction

Diabetes mellitus, a chronic metabolic disease caused by defective insulin secretion and/or insufficient insulin utilization, is one of the leading causes of death and disability worldwide (1). Diabetes, as an independent risk factor for cardiovascular disease (CVD), significantly increases the risk of events such as myocardial infarction, heart failure, and stroke. Studies have shown that the risk of CVD in the diabetic population is 2–4 times higher than in the non-diabetic population (2). Against the backdrop of accelerating aging in China, the population of middle-aged and elderly diabetic patients is growing, and their cardiovascular health management has become a significant public health challenge (3, 4). Notably, although the control of traditional risk factors (e.g., hypertension, dyslipidemia) has gradually improved, the burden of diabetes-related CVD has not been significantly reduced (5). Therefore, it is crucial to explore novel biomarkers to optimize risk stratification strategies and boost the identification of people at high cardiovascular risk for diabetes.

Insulin resistance (IR), as a core pathological feature of type 2 diabetes mellitus, has been widely confirmed as an independent risk factor for the development of CVD (6, 7). In recent years, the triglyceride-glucose (TyG) index has received widespread attention as a convenient and reliable surrogate for IR, and its strong predictive ability for CVD and CVD-associated risk factors has been confirmed in several studies (8–10). On the other hand, obesity, especially central obesity, is a dominant driver of IR and CVD (11). As a new type of body fat distribution index, the body roundness index (BRI), calculated from waist circumference (WC) and height, can more accurately reflect the degree of central obesity and visceral fat accumulation, and more accurately reflect the metabolic risk associated with obesity than the body mass index (BMI) (12). Previous studies have shown that BRI is strongly associated with IR, hypertension, and metabolic syndrome, and has independent predictive value for CVD risk (13–15).

It is worth noting that in diabetic patients, IR and central obesity often coexist and promote each other, forming a “metabolic vicious circle” and further exacerbating the risk of CVD (16). The combination of the TyG index (reflecting metabolic IR) and BRI (reflecting fat distribution abnormalities) can portray the metabolic disorder status of diabetic patients more comprehensively and predict their CVD risk more accurately in a different dimension. Metabolic disorders in diabetic patients from different dimensions may more accurately predict the risk of CVD. However, most of the current studies focus on a single indicator or the general population, and there is a lack of in-depth investigation on the predictive value of the combination of the two in diabetes mellitus, which is a high-risk group. To fill this knowledge gap, the present study utilized data from the China Health and Retirement Longitudinal Study (CHARLS) 2011–2018, a large sample of prospective cohort data, to investigate the individual and joint associations of the TyG index and BRI with CVD risk in middle-aged and elderly diabetic populations.

## 2 Methods

### 2.1 Study design and population

This study utilizes data from the CHARLS, a nationwide cohort study focusing on the middle-aged and elderly population in China. For detailed information on research design, please refer to the published literature (17, 18). The first national baseline survey, initiated in June 2011, employed multistage stratified probability sampling to recruit 17,708 participants aged 45 and above and their spouses from 150 counties across 28 provinces. They were then followed up every 2 to 3 years. Among them, blood samples from participants were collected in 2011 and 2015, and uniformly sent to the central laboratory (You’anmen Clinical Laboratory Center of Capital Medical University) for detailed analysis. The CHARLS study was approved by the Peking University Institutional Review Board (IRB00001052-11015), and all participants submitted informed consent when they joined.

**Figure 1:**
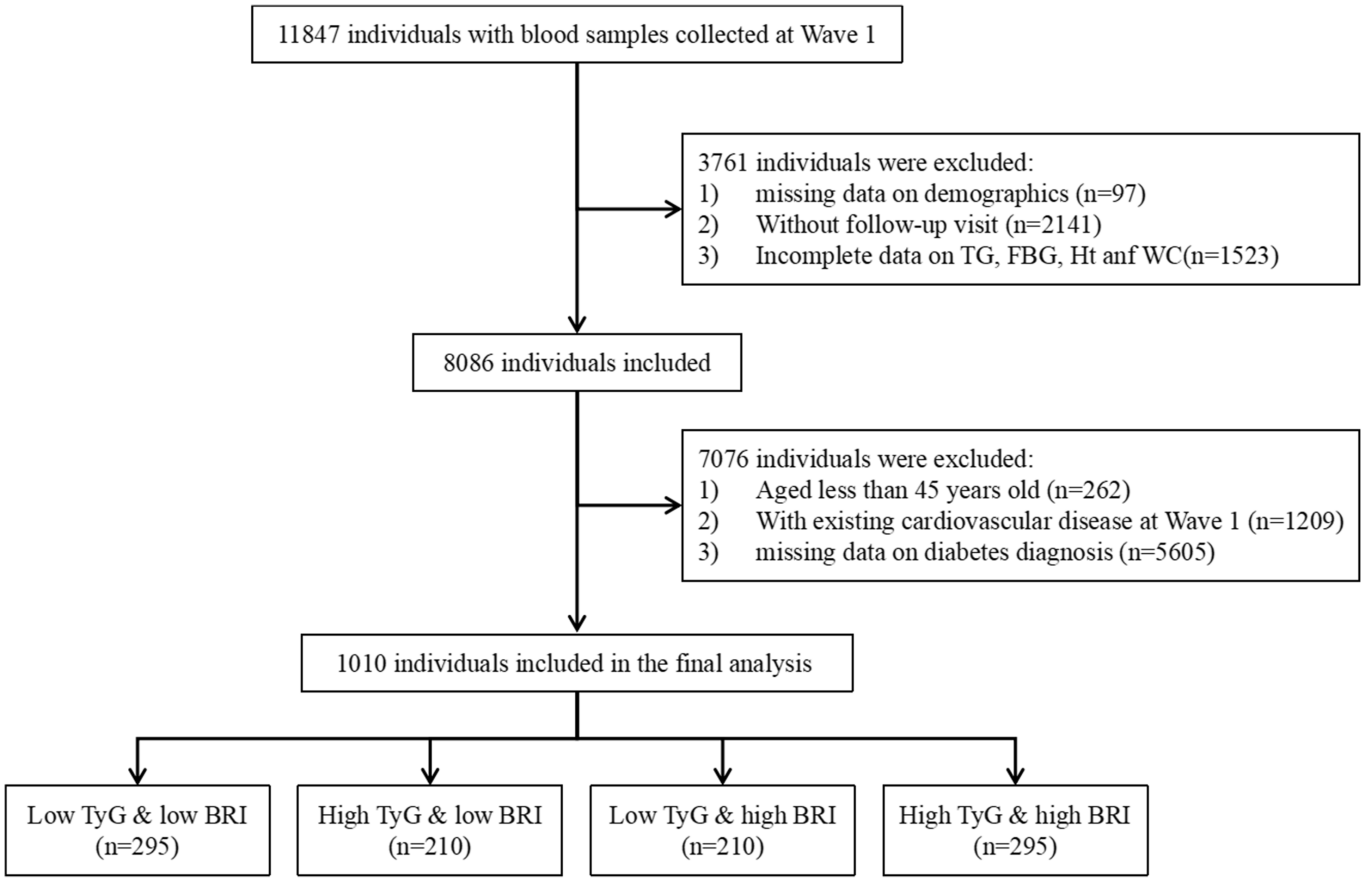
Flowchart of the study population. TyG, triglyceride glucose; BMI, body mass index; TG, triglyceride; FBG, fasting blood glucose; Ht, height; Wc, waist circumference.

Our study used the CHARLS data from 2011 to 2018. Data from Wave 1 (2011) were identified as baseline data. We first included 11,847 participants with serologic testing data recorded at Wave 1 and excluded participants with no demographic data (*n* = 97), loss to follow up (*n* = 214), and missing TyG index and BRI baseline data (*n* = 1,523). Second, we excluded participants who were younger than 45 years (*n* = 262), had a previous heart attack, stroke, or received the corresponding medication (*n* = 1,209), and had no previous diabetes (*n* = 5,605). Finally, we identified a total of 1,010 participants for our analysis (Figure 1).

### 2.2 Definition of diabetes

Diabetes was defined as meeting any of the following criteria (1): (1) self-reported physician-diagnosed diabetes; (2) current use of any anti-diabetic medication or insulin; (3) fasting blood glucose (FBG) ≥126 mg/dL (7.0 mmol/L) or glycated hemoglobin (HbA1c) ≥6.5%. FBG and HbA1c were both collected from participants’ serum at baseline and analyzed uniformly by the central laboratory using enzyme colorimetry and boronated affinity high-performance liquid chromatography, respectively.

### 2.3 Assessment of exposure

The exposure variables in this study were TyG index and BRI. Participants were serum sampled after 8 h of fasting at the time of enrollment in 2011. Blood samples were stored at 4 °C immediately after collection and transported to Beijing for testing and analysis within the specified time frame. Total triglyceride (TG) and FPG concentrations were accurately determined using an enzyme colorimetric method. The TyG index was calculated using the formula: TyG index = ln [TG (mg/dL) × FPG (mg/dL)/2] (19). Participants stood barefoot on a range finder to measure their height. WC is measured at the navel level when the participant is standing. BRI was calculated using the formula: $\mathrm{BRI}=364.2-365.5\times \sqrt{1-{\frac{\left(\frac{\mathrm{WC}}{2\pi }\right)}{{\left(0.5\times H\right)}^{2}}}^{2}}$ , where WC denotes waist circumference (cm) and H denotes height (cm) (20).

### 2.4 Assessment of outcomes

CVD occurrence acts as the endpoint event for this study. Participants enrolled in the study were inquired “Have you been diagnosed with a stroke/heart condition (including heart attack, coronary heart disease, angina, congestive heart failure or other heart problems) by a doctor?” and “Are you now undergoing any of the following treatments (Taking Chinese Traditional Medicine/Taking Western Modern Medicine/Other treatments/None of the Above) to treat stroke/heart condition or its complications?” A “yes” answer to any of the above three questions will result in a person being diagnosed with CVD.

### 2.5 Covariates

Covariates were derived from the first wave of the CHARLS data, collected by trained professionals through face-to-face interviews using structured questionnaires. The covariates in this study contained the following data: (1) demographic data: age, sex, residence, marital status, and education level; (2) body measurements: BMI, systolic blood pressure (SBP) and diastolic blood pressure (DBP); (3) lifestyle: smoking and drinking status; (4) medical history: psych problem, dyslipidemia, hypertension, liver disease, lung disease, kidney disease, digestive disorders, and cancer; (5) treatments: dyslipidemia and hypertension medications. (6) Laboratory tests: HbA1c, FPG, TG, total cholesterol (TC), high-density lipoprotein cholesterol (HDL-C), low-density lipoprotein cholesterol (LDL-C), C-reactive protein (CRP), serum creatinine (Scr), blood urea nitrogen (BUN), and uric acid (UA). Patients with hypertension were defined as those with a self-reported history of hypertension or those who were receiving specific treatment for hypertension, or those who had a mean SBP of ≥140 mmHg or a mean DBP of ≥90 mmHg at baseline (21). Dyslipidemia is defined as self-reported history of dyslipidemia, use of lipid-lowering medications, or laboratory tests showing TG ≥150 mg/dL, TC ≥240 mg/dL, HDL-C <40 mg/dL, or LDL-C ≥160 mg/dL (22).

### 2.6 Statistical analysis

We used the Kolmogorov–Smirnov test and Levene’s test to assess the normality of distribution and homogeneity of variance of continuous variables, respectively. Continuous variables that conformed to normal distribution were expressed as mean and standard deviation (SD). Continuous variables that do not follow a normal distribution were described by median and interquartile range (IQR). Categorical variables were described using frequencies and percentages. Where appropriate, baseline characteristics were compared between groups using chi-square tests, analysis of variance (ANOVA), or Kruskal–Wallis rank sum tests. Based on previous studies (23, 24), we used the median values of the TyG index (9.22) and BRI (4.52) as cutoffs to categorize respondents into four groups: low TyG & low BRI, high TyG & low BRI, low TyG & high BRI, and high TyG & high BRI.

For the missing data items, we assume that they are randomly missing and are solved by Multiple Imputation by Chained Equations. Detailed missing data information is described in Supplementary Table S1.

To assess the incidence of cardiovascular events, we calculated the incidence per 1,000 person-years for each outcome. Cox proportional risk regression models were used to estimate hazard ratio (HR) and 95% confidence interval (CI) for outcomes by group. Three models were estimated: in model 1, adjusted for age and sex; in model 2, variables in model 1 were adjusted as well as for smoking status, drinking status, marital status, education level, and place of residence; and in model 3, variables in model 2 plus CRP, LDL-C, TC, UA, Scr.

Using restricted cubic splines (RCS), we investigated the potential nonlinear associations between the TyG index with CVD risk and between BRI with CVD risk. To further test the potential association of the TyG index and BRI with the occurrence of CVD in the diabetic population, we performed joint association analysis, multiplicative and additive interaction analysis. And we performed subgroup analyzes based on different age groups, sex, smoking status, drinking status, and individuals with hypertension and dyslipidemia. For the additive interaction analysis, we calculated the relative excess risk due to interaction (RERI), the synergy index (SI), and the proportion attributable to interaction (AP).

### 2.7 Sensitivity analyzes

Sensitivity analyzes were performed to test the robustness of the results. First, we fitted a Cox regression model that excluded participants with any missing variables to eliminate the possible effect of missing values on the primary outcome. Besides, to strengthen the robustness of the primary outcome, we applied the cumulative TyG index and cumulative BRI for baseline and subgroup analyzes. In addition, we used logistic proportional risk regression models to estimate the odds ratio (OR) and 95% confidence interval (CI) for the association of the cumulative TyG index and cumulative BRI with the occurrence of CVD in the diabetic population. The same three models mentioned above were developed. The outcome event was defined as the incidence of CVD in diabetic patients in 2018. The cumulative TyG index and cumulative BRI were calculated using the formula: (TyG index or BRI score 2011 + TyG index or BRI score 2015)/2 × time (2015–2012).

We used R language software version 4.5.0 for all analyzes. We consider a *p*-value of less than 0.05 to indicate a significant difference.

## 3 Results

### 3.1 Baseline characteristics of participants

A total of 1,010 patients, 471 (46.6%) males and 539 (53.4%) females, with a mean age of 59.01 ± 8.64 years, were included in this study. Participants were categorized into four subgroups based on TyG and BRI levels: low TyG & low BRI (*n* = 295), high TyG & low BRI (*n* = 210), low TyG & high BRI (*n* = 210), and high TyG & high BRI (*n* = 295). The baseline characteristics of the enrolled participants are shown in Table 1. The results showed that the low TyG & high BRI group was the oldest (60.59 ± 9.22 years), and the high TyG & high BRI group had the highest percentage of females (69.5%). Crucially, the high TyG & high BRI group were at the highest levels of almost all cardiovascular metabolic risk indicators (including glucose, lipids, blood pressure, CRP, etc.), accompanied by the highest prevalence of dyslipidemia and hypertension and rates of medication use, indicating the most severe combined metabolic risk. In contrast, the low TyG & low BRI groups had relatively optimal indicators. There were no statistically significant differences between the groups in terms of educational level, marital status, residence, psych problem, and prevalence of liver and kidney diseases (*p*-value >0.05).

**Table 1:** Baseline characteristics of participants.

Characteristics	All	Low TyG & low BRI	High TyG & low BRI	Low TyG & high BRI	High TyG & high BRI	*p*-value
*n*	1,010	295	210	210	295	
Age, years, mean (SD)	59.01 (8.64)	59.37 (8.90)	57.89 (7.85)	60.59 (9.22)	58.33 (8.33)	0.005
Sex (female), *n* (%)	539 (53.4)	113 (38.3)	90 (42.9)	146 (69.5)	190 (64.4)	<0.001
Current married, *n* (%)	858 (85.0)	248 (84.1)	176 (83.8)	171 (81.4)	263 (89.2)	0.089
Education level, *n* (%)						0.214
Elementary school or below	273 (27.0)	71 (24.1)	67 (31.9)	52 (24.8)	83 (28.1)	
Middle school	707 (70.0)	219 (74.2)	134 (63.8)	151 (71.9)	203 (68.8)	
College or above	30 (3.0)	5 (1.7)	9 (4.3)	7 (3.3)	9 (3.1)	
Residence, *n* (%)						0.303
Urban	167 (16.5)	40 (13.6)	38 (18.1)	41 (19.5)	48 (16.3)	
Rural	843 (83.5)	255 (86.4)	172 (81.9)	169 (80.5)	247 (83.7)	
Smoking status, *n* (%)						<0.001
Current smoker	298 (29.6)	120 (40.7)	77 (37.0)	37 (17.7)	64 (21.7)	
Former smoker	89 (8.8)	21 (7.1)	23 (11.1)	22 (10.5)	23 (7.8)	
Never smoked	620 (61.6)	154 (52.2)	108 (51.9)	150 (71.8)	208 (70.5)	
Drinking status, *n* (%)						<0.001
Current drinker	323 (32.0)	108 (36.6)	88 (42.1)	45 (21.4)	82 (27.8)	
Former drinker	94 (9.3)	35 (11.9)	16 (7.7)	21 (10.0)	22 (7.5)	
Never drinker	592 (58.7)	152 (51.5)	105 (50.2)	144 (68.6)	191 (64.7)	
Psych problem, *n* (%)	7 (0.7)	1 (0.3)	1 (0.5)	4 (1.9)	1 (0.3)	0.193
Dyslipidemia, *n* (%)	143 (14.3)	15 (5.1)	21 (10.2)	30 (14.6)	77 (26.2)	<0.001
Hypertension, *n* (%)	322 (32.0)	47 (15.9)	54 (25.8)	98 (46.7)	123 (42.1)	<0.001
Kidney disease, *n* (%)	43 (4.3)	12 (4.1)	12 (5.7)	6 (2.9)	13 (4.4)	0.548
Liver disease, *n* (%)	31 (3.1)	9 (3.1)	4 (1.9)	7 (3.3)	11 (3.8)	0.726
Lunge disease, *n* (%)	79 (7.9)	30 (10.2)	11 (5.3)	14 (6.7)	24 (8.2)	0.212
Digestive disease, *n* (%)	199 (19.7)	65 (22.0)	36 (17.2)	42 (20.0)	56 (19.0)	0.593
Cancer, *n* (%)	11 (1.1)	1 (0.3)	3 (1.4)	1 (0.5)	6 (2.0)	0.197
Dyslipidemia medications, *n* (%)	80 (8.0)	7 (2.4)	11 (5.4)	19 (9.2)	43 (14.7)	<0.001
Hypertension medications, *n* (%)	239 (23.8)	29 (9.8)	37 (17.7)	73 (34.8)	100 (34.2)	<0.001
BMI, kg/m^2^, median (IQR)	24.25 [21.88, 26.95]	21.74 [20.03, 23.29]	22.84 [21.22, 24.59]	26.66 [24.46, 28.49]	26.69 [24.85, 28.91]	<0.001
SBP, mmHg, mean (SD)	132.47 (20.63)	127.35 (19.90)	129.56 (20.40)	135.95 (21.89)	137.24 (19.08)	<0.001
DBP, mmHg, mean (SD)	76.59 (11.29)	73.09 (10.50)	75.89 (11.55)	77.26 (10.83)	80.12 (11.11)	<0.001
HbA1c, %, median (IQR)	5.50 [5.10, 6.60]	5.30 [5.00, 5.80]	5.40 [5.10, 6.90]	5.60 [5.20, 6.40]	6.00 [5.40, 7.48]	<0.001
FPG, mg/dL, median (IQR)	140.85 [128.88, 173.70]	134.10 [126.36, 148.50]	152.82 [136.58, 209.25]	129.42 [111.33, 140.49]	167.04 [139.95, 220.77]	<0.001
TG, mg/dL, median (IQR)	138.06 [92.04, 229.21]	85.85 [65.93, 107.97]	219.04 [160.18, 354.44]	103.99 [86.07, 123.68]	234.52 [182.31, 344.26]	<0.001
TC, mg/dL, median (IQR)	196.01 [170.49, 226.84]	183.25 [160.44, 212.05]	202.00 [177.93, 234.67]	196.20 [169.43, 219.49]	206.83 [177.26, 239.69]	<0.001
HDL-C, mg/dL, median (IQR)	44.46 [35.57, 54.51]	52.19 [43.69, 63.40]	40.01 [32.09, 50.35]	48.33 [41.75, 55.96]	36.34 [30.15, 44.46]	<0.001
LDL-C, mg/dL, mean (SD)	113.73 (40.87)	116.44 (33.90)	103.63 (43.85)	127.43 (34.04)	108.24 (46.50)	<0.001
CRP, mg/L, median (IQR)	1.24 [0.64, 2.63]	0.76 [0.46, 1.83]	1.14 [0.66, 2.25]	1.37 [0.73, 2.42]	1.76 [0.94, 3.59]	<0.001
Scr, mg/dL, median (IQR)	0.76 [0.65, 0.88]	0.77 [0.64, 0.89]	0.77 [0.66, 0.91]	0.73 [0.64, 0.86]	0.76 [0.66, 0.87]	0.236
BUN, mg/dL, median (IQR)	15.41 [12.94, 18.46]	15.20 [12.56, 18.90]	15.64 [13.24, 18.44]	15.66 [13.06, 18.91]	15.21 [12.94, 17.51]	0.373
UA, mg/dL, mean (SD)	4.54 (1.33)	4.31 (1.20)	4.69 (1.41)	4.47 (1.31)	4.69 (1.39)	0.001
TyG, mean (SD)	9.35 (0.85)	8.63 (0.44)	9.96 (0.59)	8.75 (0.36)	10.06 (0.65)	<0.001
BRI, median (IQR)	4.52 [3.60, 5.56]	3.48 [2.82, 4.00]	3.73 [3.27, 4.23]	5.59 [4.91, 6.21]	5.52 [4.99, 6.30]	<0.001

Values are mean (SD), median (IQR), or *n* (%).

BMI, body mass index; SBP, systolic blood pressure; DBP, diastolic blood pressure; HbA1c, glycated hemoglobin; FPG, fasting plasma glucose; TG, triglycerides; TC, total cholesterol; HDL-C, high-density lipoprotein cholesterol; LDL-C, low-density lipoprotein cholesterol; CRP, C-reactive protein; Scr, serum creatinine; BUN, blood urea nitrogen; UA, uric acid; TyG, triglyceride glucose; BRI, body roundness index; IQR, interquartile range; SD, standard deviation.

### 3.2 Association of the TyG index and BRI with CVD risk

A total of 251 (24.9%) middle-aged and elderly persons with diabetes developed new-onset CVD during the maximum 7-year follow-up period. To explore the association between the TyG index and BRI with CVD risk, we adjusted three Cox proportional risk regression models, as detailed in Table 2. After adjusting for multiple covariates (Model 3), participants with high TyG index had a 39% (95% CI 1.06–1.83) increased risk of CVD when compared to low TyG index; when compared to low BRI, the participants with high BRI had a 73% (95% CI 1.32–2.27) increased risk of CVD. In contrast, when using low TyG & low BRI as a reference, participants with low TyG & high BRI and high TyG & high BRI had an 85% (95% CI 1.25–2.73) and 123% (95% CI 1.53–3.24) increased risk of CVD, respectively. Interestingly, there was no significant difference in CVD risk between participants with high TyG & low BRI compared to those with low TyG & low BRI in any model (*p*-value >0.05).

**Table 2:** Association of the TyG index and BRI and the risk of CVD incidence.

COX	Case	Incidence rate^a^ (95% CI)	Crude model		Model 1		Model 2		Model 3	
			HR (95% CI)	*p*-value	HR (95% CI)	*p*-value	HR (95% CI)	*p*-value	HR (95% CI)	*p*-value
TyG										
Low TyG	110	2.8 (2.3–3.4)	Ref.		Ref.		Ref.		Ref.	
High TyG	141	3.7 (3.1–4.4)	1.34 (1.04–1.72)	0.022	1.33 (1.04–1.71)	0.024	1.31 (1.02–1.68)	0.037	1.39 (1.06–1.83)	0.019
BRI										
Low BRI	94	2.4 (1.9–2.9)	Ref.		Ref.		Ref.		Ref.	
High BRI	157	4.2 (3.5–4.9)	1.83 (1.42–2.36)	<0.001	1.77 (1.36–2.31)	<0.001	1.68 (1.29–2.20)	<0.001	1.73 (1.32–2.27)	<0.001
TyG and BRI										
Low TyG & low BRI	48	2.1 (1.5–2.7)	Ref.		Ref.		Ref.		Ref.	
High TyG & low BRI	46	2.8 (2.1–3.8)	1.42 (0.95–2.13)	0.089	1.42 (0.95–2.13)	0.089	1.34 (0.89–2.02)	0.155	1.44 (0.95–2.20)	0.087
Low TyG & high BRI	62	4.0 (3.0–5.1)	2.03 (1.39–2.96)	<0.001	1.95 (1.32–2.86)	<0.001	1.78 (1.21–2.62)	0.005	1.85 (1.25–2.73)	0.002
High TyG & high BRI	95	4.3 (3.5–5.3)	2.22 (1.57–3.14)	<0.001	2.16 (1.51–3.08)	<0.001	2.04 (1.43–2.91)	<0.001	2.23 (1.53–3.24)	<0.001

^a^Per 1,000 person-years.

Crude model: we did not adjust for other covariates.

Model 1: we adjusted for age and sex.

Model 2: we adjusted for age, sex, smoking status, drinking status, marital status, education level, and residence.

Model 3: we adjusted for age, sex, smoking status, drinking status, marital status, education level, residence, CRP, LDL-C, TC, UA, Scr.

TyG, triglyceride glucose; BRI, body roundness index; CRP, C-reactive protein; LDL-C, low-density lipoprotein cholesterol; TC, total cholesterol; UA, uric acid; Scr, serum creatinine; HR, hazard ratio; Ref, reference; CI, confidence interval.

**Figure 2:**
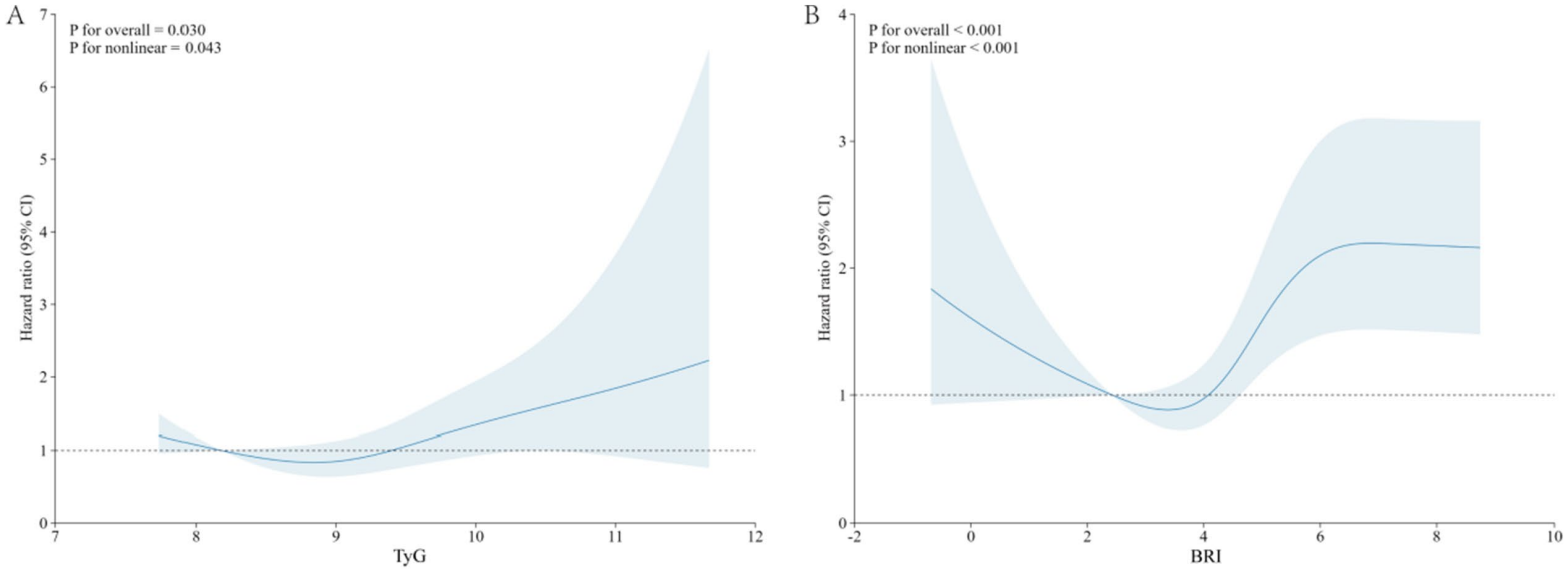
RCS curve of the TyG index and BRI with the prevalence of CVD in middle-aged and elderly diabetic patients. **(A)** TyG index; **(B)** BRI. The dark blue solid line indicates the estimated effect, and the light blue shaded area indicates the 95% confidence interval (CI).

RCS analysis indicate that both the TyG index (*p* for overall = 0.030, *p* for nonlinear = 0.043) and BRI (*p* for overall <0.001, *p* for nonlinear <0.001) exhibit a nonlinear relationship with the risk of new-onset CVD in middle-aged and elderly diabetic patients. When the TyG index >8.649 or BRI >3.613, it exhibits a positive correlation with CVD risk in middle-aged and elderly diabetic populations. Otherwise, it is negatively correlated (Figure 2).

### 3.3 Interaction between the TyG index and BRI

There was no statistically significant additive or multiplicative interaction between the TyG index and BRI in increasing the risk of CVD in middle-aged and elderly persons with diabetes when adjusted for different confounders (Table 3).

**Table 3:** Interaction between the TyG index and BRI on cardiovascular disease risk.

Interactive indices	Interactive effects (95% CI)		
	Model 1	Model 2	Model 3
Additive effect			
RERI	−0.21 (−1.05–0.63)	−0.13 (−0.85–0.59)	−0.08 (−0.73–0.57)
AP	−0.10 (−0.49–0.29)	−0.08 (−0.49–0.34)	−0.05 (−0.50–0.39)
SI	0.85 (0.32–1.37)	0.84 (0.12–1.57)	0.85 (−0.21–1.91)
Multiplicative effect	0.78 (0.47–1.31)	0.85 (0.51–1.43)	0.91 (0.54–1.54)

Model 1: we adjusted for age and sex.

Model 2: we adjusted for age, sex, smoking status, drinking status, marital status, education level, and residence.

Model 3: we adjusted for age, sex, smoking status, drinking status, marital status, education level, residence, CRP, LDL-C, TC, UA, Scr.

RERI, relative excess risk due to interaction; AP, proportion attributable to interaction; SI, synergy index; CI, confidence interval.

### 3.4 Subgroup analyzes

To further explore the relationship between the TyG index and BRI and the risk of new-onset CVD, we conducted a series of subgroup analyzes. The results showed that the combined effect of the TyG index and BRI on CVD risk in persons with diabetes was independent of age, sex, smoking status, drinking status, and hypertension, except for dyslipidemia (*p* for interaction = 0.003) (Table 4). In addition, we observed that the BRI continuous variable interacted with gender, age, smoking status, drinking status, marital status, and place of residence in terms of CVD risk (*p* for interaction <0.05) (Supplementary Figure S1). However, the above results were not found in the TyG index (Supplementary Figure S2).

**Table 4:** Subgroup analysis of the correlation between the TyG index and BRI on CVD risk.

Characteristics	Low TyG & low BRI	High TyG & low BRI	Low TyG & high BRI	High TyG & high BRI	*p*-value
Age					0.256
<60	Ref	1.28 (0.70–2.33)	1.77 (0.97–3.22)	1.66 (0.96–2.90)	
≥60	Ref	1.13 (0.62–2.05)	1.09 (0.61–1.94)	1.35 (0.78–2.33)	
Sex					0.2
Male	Ref	0.84 (0.47–1.52)	1.22 (0.64–2.30)	1.47 (0.83–2.59)	
Female	Ref	1.73 (0.94–3.21)	1.44 (0.81–2.58)	1.47 (0.84–2.60)	
Drinking status					0.27
Current drinker	Ref	1.04 (0.49–2.17)	1.99 (0.90–4.41)	1.60 (0.77–3.32)	
Former drinker	Ref	0.69 (0.18–2.62)	0.49 (0.12–1.96)	1.78 (0.51–6.22)	
Never drinker	Ref	1.29 (0.73–2.30)	1.32 (0.78–2.26)	1.39 (0.84–2.32)	
Smoking status					0.152
Current smoker	Ref	0.83 (0.36–1.88)	1.67 (0.72–3.91)	2.23 (1.07–4.64)	
Former smoker	Ref	0.56 (0.15–2.11)	1.20 (0.38–3.77)	0.72 (0.20–2.66)	
Never smoked	Ref	1.81 (1.03–3.17)	1.39 (0.81–2.39)	1.42 (0.85–2.36)	
Dyslipidemia					0.003
No	Ref	1.36 (0.87–2.14)	1.61 (1.03–2.52)	1.62 (1.05–2.48)	
Yes	Ref	0.34 (0.10–1.12)	0.28 (0.09–0.84)	0.45 (0.17–1.20)	
Hypertension					
No	Ref	1.31 (0.78–2.18)	1.34 (0.77–2.31)	1.42 (0.87–2.31)	0.795
Yes	Ref	1.25 (0.58–2.68)	1.42 (0.70–2.88)	1.73 (0.86–3.47)	

TyG, triglyceride glucose; BRI, body roundness index; Ref, reference.

### 3.5 Sensitivity analyzes

To test the robustness of the findings, we applied several validation methods, all of which yielded consistent conclusions. First, after excluding participants with missing values, the main results of the baseline (Supplementary Table S2), Cox proportional risk regression model (Supplementary Table S3), and subgroup analyzes (Supplementary Table S4) remained consistent with the previous ones. Second, we used the cumulative 2011 to 2015 values of the TyG index and BRI for the combined subgroups, and not only were there no significant changes in the baseline primary outcomes from the previous period (Supplementary Table S5), but the results of the logistic regression model analyzes also showed that the associations of the TyG index and the BRI individually and in combination on the risk of CVD remained unchanged (Supplementary Table S6). Finally, in subgroup analyzes, adjusting the cumulative variables for both indices did not affect the primary outcome and still supports a significant effect of dyslipidemia on the combined effect of the TyG index and BRI on CVD risk (Supplementary Table S7).

## 4 Discussion

Based on data from the CHARLS cohort study, this study investigated the association between the TyG index and BRI on CVD risk in middle-aged and elderly persons with diabetes. The results showed that high TyG index and high BRI were significantly associated with elevated CVD risk in a representative sample of 1,010 individuals. The highest risk of CVD was observed in participants with both high TyG index and high BRI after adjusting for confounders, when the median TyG index (9.22) and BRI (4.52) were used as the cut-off points for the joint grouping. This risk was 123% higher than in participants with both low TyG index and low BRI. Although no significant interaction was observed in this study, this composite indicator may serve as a valuable supplement to existing risk assessment tools, guiding the development of personalized cardiovascular disease prevention strategies for diabetic patients.

The present study confirms that the TyG index (a surrogate marker reflecting IR) is significantly and positively associated with CVD risk in persons with diabetes, which is consistent with previous studies (10, 25). Mechanistically, the high TyG index suggests that decreased efficiency of glucose uptake by peripheral tissues leads to IR, which in turn promotes endothelial inflammation, causes lipid metabolism disorders, exacerbates oxidative stress, and ultimately accelerates the formation and progression of atherosclerosis (8). Several studies have confirmed that the TyG index is an independent risk factor for CVD (8, 10). A meta-study showed that the high TyG index was significantly associated with a 95 and 26% increased risk of coronary artery disease and stroke (26). For persons with diabetes and pre-diabetes, an NHANES-based cohort study focusing on people under 65 years of age in the United States showed that the high TyG index was nonlinearly associated with the risk of CVD (27). A cohort study based on Chinese middle-aged and older persons with diabetes showed that the high TyG index led to a 123.4% increased risk of CVD after adjusting for confounders (28). Together, these studies emphasize the importance of the TyG index as a predictive marker of CVD risk, a finding that is particularly strong in persons with diabetes. The results of our study showed that participants with the TyG index greater than 9.22 had a 39% increased CVD risk. This result affirms the predictive value of the TyG index for CVD risk in middle-aged and older persons with diabetes.

BRI, an emerging anthropometric indicator, accurately reflects the degree of visceral fat accumulation by quantifying the geometric relationship between WC and height (12). A prospective cohort study involving 9,935 middle-aged and elderly participants showed that BRI can be used as a predictor of CVD, and a high BRI was associated with a 55% increased risk of CVD. A cohort study by Zhou et al. (29) demonstrated a U-shaped association of BRI with cardiovascular mortality and all-cause mortality, a result superior to that predicted by WC and a body shape index (ABSI). Interestingly, the cohort study by Kong et al. (30) focused on the role of BRI in the risk of new-onset CVD in middle-aged persons with and without diabetes. While the results showed a significant association between BRI and CVD risk in the group without diabetes, its mediating effect on new-onset CVD in persons with diabetes was particularly pronounced in the long term (30). Our findings suggest a nonlinear association between BRI and CVD, with middle-aged and older participants with a BRI greater than 4.52 having a 73% increased risk of CVD. And there was a significant interaction between BRI and gender, age, smoking status, drinking status, marital status, and place of residence in terms of CVD risk. Therefore, accurate assessment of visceral adiposity or central obesity has significant clinical value in predicting CVD risk.

IR and lipid metabolism disorders have a vicious circle relationship. This mechanism is particularly prominent in metabolic diseases such as diabetes and obesity. The core pathophysiological changes are the abnormal distribution and dysfunction of adipose tissue (especially visceral fat). Specifically, in the IR state, hormone-sensitive lipase activity is enhanced to promote lipolysis, leading to the breakdown of large amounts of TG into free fatty acids (FFAs) and glycerol and their release into the blood (31). The excessive FFAs further inhibit tyrosine phosphorylation of insulin receptor substrates (IRS), exacerbating IR and creating a self-reinforcing cycle of metabolic disorders (32). Notably, visceral fat plays a key role in this process. As a highly metabolically active tissue, its release of FFAs and inflammatory factors not only exacerbates IR but also promotes very low-density lipoprotein (VLDL) synthesis, which contributes to the development of atherosclerosis (33). Previous studies have demonstrated the interaction between the TyG index and traditional obesity indicators (e.g., BMI, WC, etc.) for the prediction of CVD morbidity and mortality in the general population (34, 35). However, fewer studies have been conducted on the relationship between IR and central obesity. Wang’s et al. (23) study involving 6,621 middle-aged and older adults demonstrated the combined effect of the TyG index and BRI on stroke risk, emphasizing the critical role of IR and visceral obesity in the prediction of stroke incidence. Bai et al. (36) used the TyG index and BRI as a combined index. And they demonstrated in a cohort study that the TyG-BRI index had a significant and independent correlation with CVD risk, and a high TyG-BRI index increased the risk of CVD events by 59.1%. Our study demonstrated a joint association of the TyG index and BRI with CVD risk in middle-aged and elderly persons with diabetes, which remained significant even after subgroup and sensitivity analyzes. Our findings showed that people with both high TyG index and high BRI diabetes had the highest incidence of CVD. The Incidence rate per 1,000 person-years was 4.3% (95% CI: 3.5–5.3), and the risk of incidence was increased by 123% compared to those with both low TyG index and low BRI. Interestingly, the presence of both the low TyG index and high BRI was associated with an 85% increased risk of CVD. In contrast, there was no significant association between the presence of both high TyG index and low BRI and the risk of CVD. It is suggested that high BRI has a greater impact on CVD risk in persons with diabetes than high TyG index. Additionally, this study acknowledges that selection bias may exist, as 1,010 diabetic patients were screened from a cohort of 17,708 individuals based on inclusion and exclusion criteria. To fully address this, we conducted a series of sensitivity analyzes and subgroup analyzes. These revealed that our findings remained consistent when participants with missing values were excluded, when participants were regrouped based on cumulative TyG index and BRI values from 2011 to 2015, or when analyzes were performed using different model specifications. Consequently, the findings demonstrate robust validity.

Although this study did not conduct direct statistical comparisons with traditional CVD risk scores (such as the Framingham or ASCVD), the combined effect of the TyG index and BRI demonstrated unique clinical value in predicting CVD risk among diabetic populations. The Framingham risk score primarily incorporates traditional risk factors, including age, blood pressure, and cholesterol, and the ASCVD risk score further includes BMI and diabetes status. Nevertheless, neither fully captures the core drivers of disease progression in middle-aged and elderly diabetic populations: insulin resistance and central obesity. The TyG index and BRI, respectively, target these two pivotal pathological pathways. Their combination provides a more comprehensive characterization of vascular disease risk associated with metabolic abnormalities in diabetic patients. Consequently, compared to conventional risk scores, this composite metric holds promise as a significant supplement for refined CVD risk stratification in diabetic populations.

This study has several limitations. Firstly, the determination of CVD outcomes relied primarily on participants’ self-reports, which may introduce recall bias and misclassification. Although this approach is feasible in large-scale cohort studies, it may lead to underreporting of asymptomatic or undiagnosed events, potentially underestimating the true risk association observed in this study. Secondly, the grouping of the TyG index and BRI is based on the sample median rather than clinically validated thresholds. While this approach has precedents in prior studies and permits comparisons of relative risk within cohorts, it may not represent the optimal cut-off point for clinical risk stratification within diabetic populations. Future research is required to establish and apply standardized, clinically relevant thresholds. Thirdly, although we examined the joint association between the TyG index and BRI for CVD, no significant additive or multiplicative interactions were detected. Nevertheless, this does not diminish the practical value of the combined TyG and BRI assessment. Fourthly, the findings of this study are based on a single CHARLS cohort. Although internal validation has confirmed the robustness of the results, they have not been validated by an independent external cohort and may harbor unidentified cohort-specific biases. Subsequent external validation is therefore particularly crucial. Finally, the study population is limited to middle-aged and elderly Chinese individuals with diabetes. Consequently, the generalizability of the conclusions to other ethnicities, geographical regions, or younger cohorts may be constrained.

## 5 Conclusion

This study found that elevated the TyG index and BRI were significantly associated with increased risk of new-onset CVD in a Chinese middle-aged and elderly persons with diabetes, and the combined assessment of the TyG index and BRI enhanced the prediction of CVD.

## Data Availability

The original contributions presented in the study are included in the article/Supplementary material, further inquiries can be directed to the corresponding author.

## Glossary

ABSI: A body shape index
AP: Attributable proportion due to interaction
ASCVD: Atherosclerotic cardiovascular disease
BMI: Body mass index
BRI: Body roundness index
BUN: Blood urea nitrogen
CHARLS: China Health and Retirement Longitudinal Study
CI: Confidence interval
CRP: C-reactive protein
CVD: Cardiovascular diseases
DBP: Diastolic blood pressure
FFAs: Free fatty acids
FPG: Fasting plasma glucose
HbA1c: Glycosylated hemoglobin A1c
HDL-C: High density lipoprotein cholesterol
HR: Hazard ratio
IQR: Interquartile range
IR: Insulin resistance
IRS: Insulin receptor substrates
LDL-C: Low density lipoprotein cholesterol
MICE: Multiple imputation by chained equations
RERI: Relative excess risk due to interaction
SBP: Systolic blood pressure
Scr: Serum creatinine
SD: Standard deviation
Ref: Reference
SI: Synergy index
TC: Total cholesterol
TG: Triglyceride
TyG: Triglyceride glucose
UA: Uric acid
VLDL: Very low-density lipoprotein
WC: Waist circumference
