# Supplementary material for: Combined association of the triglyceride glucose index and body roundness index with cardiovascular disease in middle-aged and elderly persons with diabetes: a CHARLS-based cohort study
Source: Front Nutr. 2026 Jan 14;12:1724178. doi: 10.3389/fnut.2025.1724178 (PMC12846984; doi:10.3389/fnut.2025.1724178)
Supplement: Supplementary file 1 [file Table_1.docx]

Supplementary Material

**
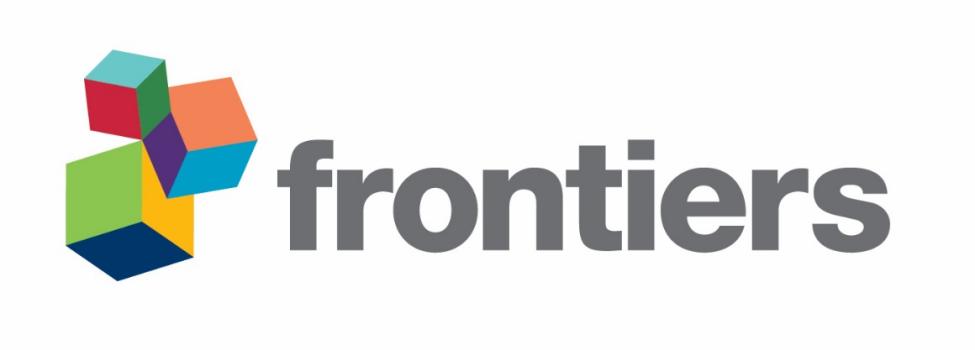
**

**Supplementary Materials**

**Fig. S1** Subgroup analysis of the correlation between BRI on CVD risk

**Fig. S2** Subgroup analysis of the correlation between TyG index on CVD risk

**Table S1** The proportion of missing covariates and imputation methods

**Table S2** Baseline characteristics after excluding participants with missing values

**Table S3** Association of TyG and BRI and the risk of CVD incidence after excluding participants with missing values

**Table S4** Subgroup analysis of the correlation between TyG index and BRI on CVD risk after excluding participants with missing values

**Table S5** Baseline characteristics grouped by cumulative TyG index and cumulative BRI

**Table S6** The correlation between cumulative TyG index and cumulative BRI with CVD incidence risk based on logistics regression model

**Table S7** Subgroup analysis of the correlation between cumulative TyG index and cumulative BRI on CVD risk**Fig. S1** Subgroup analysis of the correlation between BRI on CVD risk


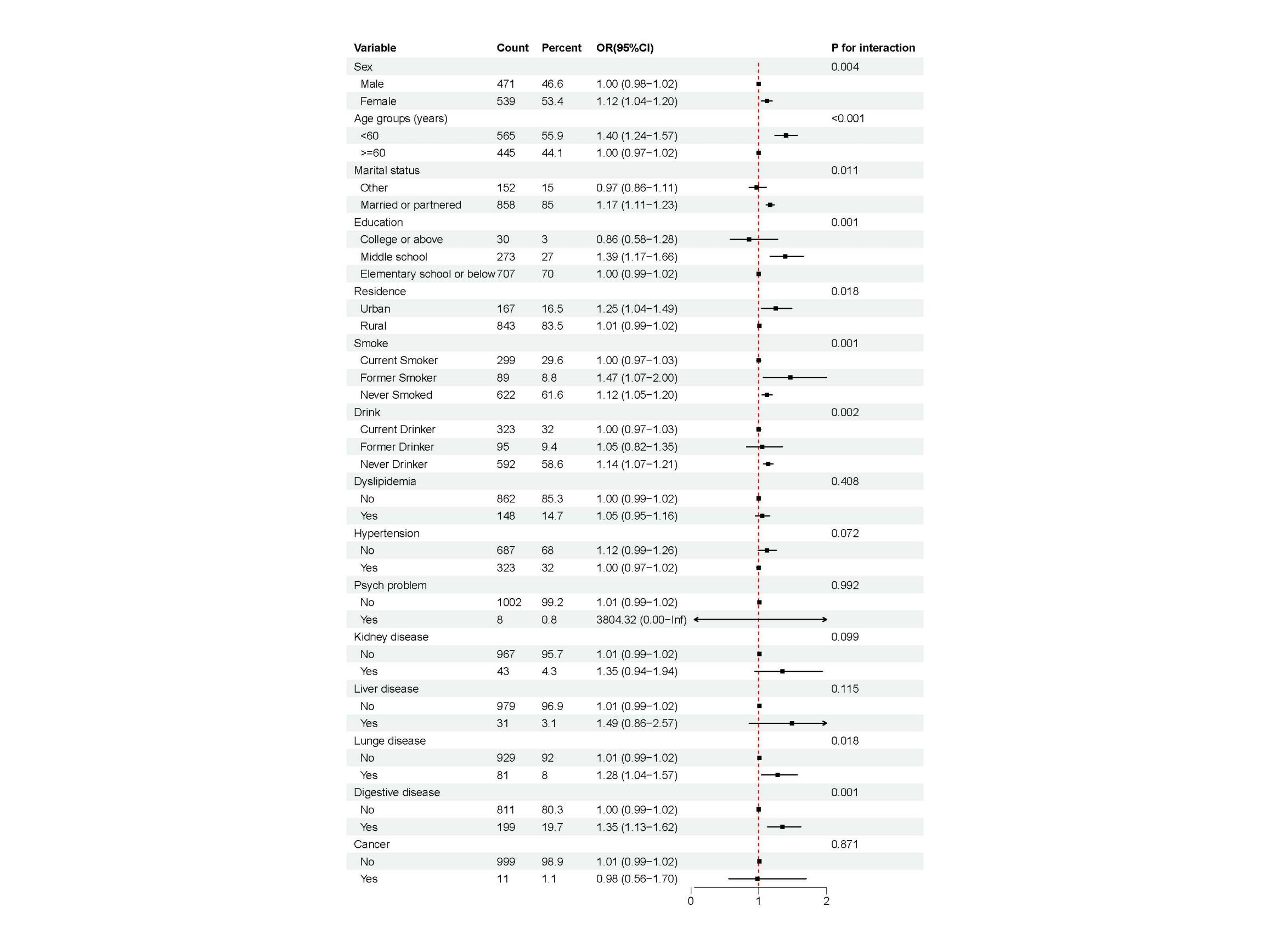


**Fig. S2** Subgroup analysis of the correlation between TyG index on CVD risk


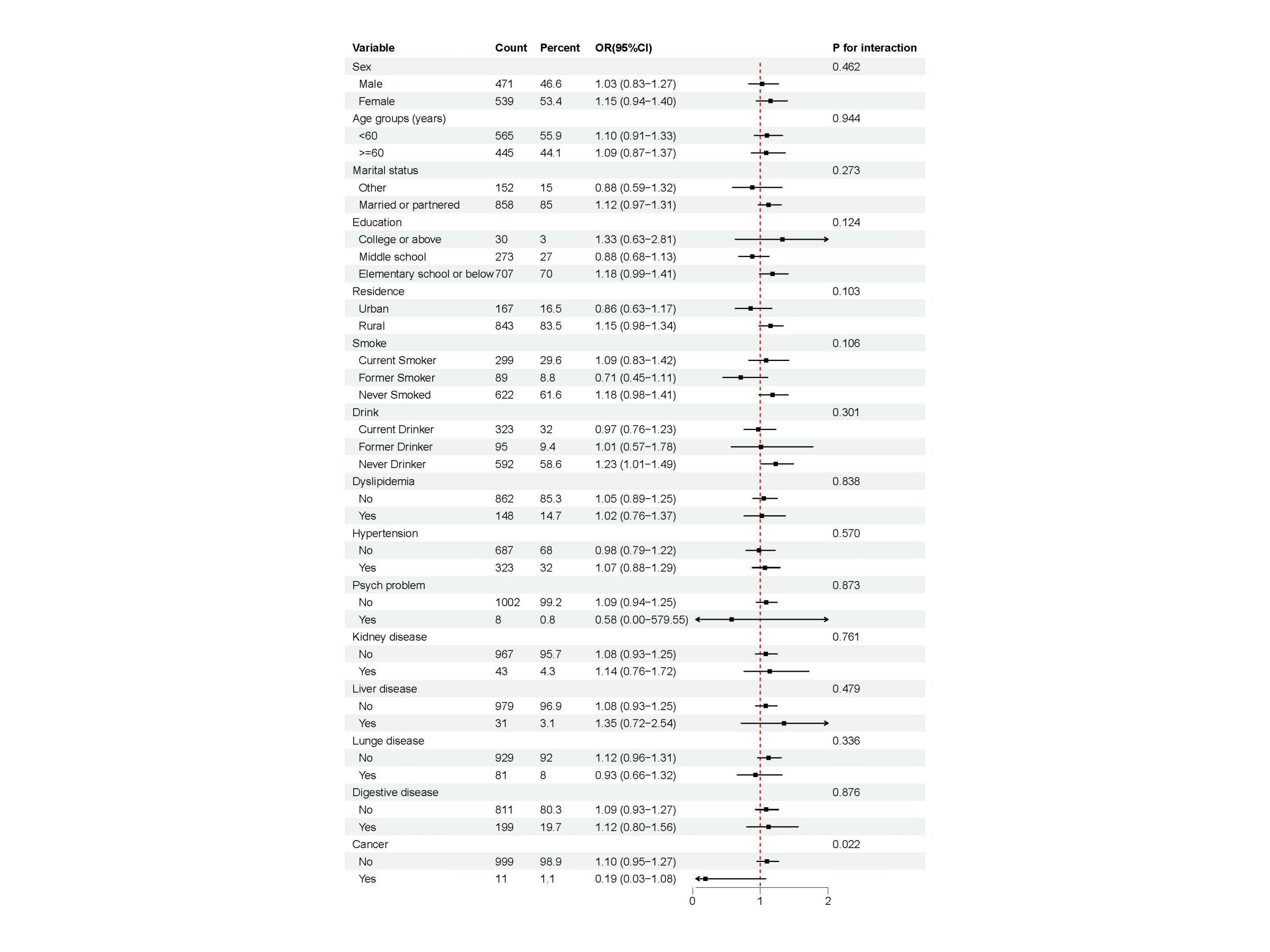


**Table S1** The proportion of missing covariates and imputation methods

| **Variable** | **Number of missing data** | **Percentage of missing data (%)** | **Imputation methods** |
| --- | --- | --- | --- |
| Smoking | 3 | 0.30 | polyreg |
| Drinking | 1 | 0.10 | polyreg |
| BMI | 5 | 0.50 | pmm |
| SBP | 8 | 0.79 | pmm |
| DBP | 8 | 0.79 | pmm |
| Psych problem | 3 | 0.30 | logreg |
| Dyslipidemia | 13 | 1.29 | logreg |
| Hypertension | 4 | 0.40 | logreg |
| Kidney disease | 4 | 0.40 | logreg |
| Liver disease | 5 | 0.50 | logreg |
| Lunge disease | 4 | 0.40 | logreg |
| Digestive disease | 1 | 0.10 | logreg |
| Cancer | 5 | 0.50 | logreg |
| Dyslipidemia medications | 14 | 1.39 | logreg |
| Hypertension medications | 4 | 0.40 | logreg |
| HbA1c | 6 | 0.59 | pmm |
| LDL-C | 11 | 1.09 | pmm |
| Scr | 3 | 0.30 | pmm |
| BUN | 1 | 0.10 | pmm |

Abbreviations: BMI: body mass index; SBP: systolic blood pressure; DBP: diastolic blood pressure; **HbA1c: glycated hemoglobin;** LDL-C: low-density lipoprotein cholesterol; **Scr: serum creatinine; BUN: blood urea nitrogenTable S2** Baseline characteristics after excluding participants with missing values

| **Characteristics** | **All** | **Low TyG & low BRI** | **High TyG & low BRI** | **Low TyG & high BRI** | **High TyG & high BRI** | **P value** |
| --- | --- | --- | --- | --- | --- | --- |
| n | 840 | 249 | 171 | 171 | 249 |  |
| Age,yearss, median (IQR) | 58.00 [53.00, 64.00] | 57.00 [53.00, 65.00] | 58.00 [52.00, 64.00] | 59.00 [54.00, 66.00] | 59.00 [51.00, 63.00] | 0.211 |
| Sex (Female), n (%) | 451 (53.7) | 95 (38.2) | 77 (45.0) | 117 (68.4) | 162 (65.1) | <0.001 |
| Current married, n (%) | 724 (86.2) | 215 (86.3) | 139 (81.3) | 145 (84.8) | 225 (90.4) | 0.061 |
| Education level, n (%) |  |  |  |  |  | 0.649 |
| Elementary school or below | 592 (70.5) | 185 (74.3) | 113 (66.1) | 121 (70.8) | 173 (69.5) |  |
| Middle school | 226 (26.9) | 59 (23.7) | 54 (31.6) | 45 (26.3) | 68 (27.3) |  |
| College or above | 22 (2.6) | 5 (2.0) | 4 (2.3) | 5 (2.9) | 8 (3.2) |  |
| Residence, n (%) |  |  |  |  |  | 0.555 |
| Urban | 136 (16.2) | 35 (14.1) | 27 (15.8) | 33 (19.3) | 41 (16.5) |  |
| Rural | 704 (83.8) | 214 (85.9) | 144 (84.2) | 138 (80.7) | 208 (83.5) |  |
| Smoking status, n (%) |  |  |  |  |  | <0.001 |
| Current Smoker | 254 (30.2) | 107 (43.0) | 65 (38.0) | 32 (18.7) | 50 (20.1) |  |
| Former Smoker | 69 (8.2) | 16 (6.4) | 19 (11.1) | 16 (9.4) | 18 (7.2) |  |
| Never Smoked | 517 (61.5) | 126 (50.6) | 87 (50.9) | 123 (71.9) | 181 (72.7) |  |
| Drinking status, n (%) |  |  |  |  |  | <0.001 |
| Current Drinker | 265 (31.5) | 94 (37.8) | 68 (39.8) | 35 (20.5) | 68 (27.3) |  |
| Former Drinker | 77 (9.2) | 29 (11.6) | 13 (7.6) | 15 (8.8) | 20 (8.0) |  |
| Never Drinker | 498 (59.3) | 126 (50.6) | 90 (52.6) | 121 (70.8) | 161 (64.7) |  |
| Psych problem, n (%) | 6 (0.7) | 1 (0.4) | 1 (0.6) | 3 (1.8) | 1 (0.4) | 0.431 |
| Dyslipidemia, n (%) | 123 (14.6) | 14 (5.6) | 13 (7.6) | 28 (16.4) | 68 (27.3) | <0.001 |
| Hypertension, n (%) | 256 (30.5) | 38 (15.3) | 40 (23.4) | 77 (45.0) | 101 (40.6) | <0.001 |
| Kidney disease, n (%) | 35 (4.2) | 11 (4.4) | 11 (6.4) | 4 (2.3) | 9 (3.6) | 0.287 |
| Liver disease, n (%) | 27 (3.2) | 7 (2.8) | 4 (2.3) | 6 (3.5) | 10 (4.0) | 0.815 |
| Lunge disease, n (%) | 60 (7.1) | 23 (9.2) | 7 (4.1) | 12 (7.0) | 18 (7.2) | 0.248 |
| Digestive disease, n (%) | 170 (20.2) | 53 (21.3) | 32 (18.7) | 34 (19.9) | 51 (20.5) | 0.937 |
| Cancer, n (%) | 7 (0.8) | 0 (0.0) | 1 (0.6) | 1 (0.6) | 5 (2.0) | 0.076 |
| Dyslipidemia medications, n (%) | 70 (8.3) | 7 (2.8) | 6 (3.5) | 17 (9.9) | 40 (16.1) | <0.001 |
| Hypertension medications, n (%) | 191 (22.7) | 24 (9.6) | 26 (15.2) | 57 (33.3) | 84 (33.7) | <0.001 |
| BMI,kg/m^2^, median (IQR) | 24.12 [21.72, 26.92] | 21.63 [20.02, 23.15] | 22.54 [20.91, 24.23] | 26.63 [24.49, 28.56] | 26.71 [24.82, 28.77] | <0.001 |
| SBP, mmHg, median (IQR) | 130.50 [117.00, 144.50] | 126.00 [113.00, 137.00] | 128.00 [116.00, 143.50] | 132.50 [118.75, 149.00] | 135.00 [123.50, 147.00] | <0.001 |
| DBP, mmHg, median (IQR) | 76.00 [68.50, 83.12] | 72.50 [66.50, 79.50] | 75.00 [67.50, 83.25] | 75.00 [70.00, 83.50] | 79.00 [72.00, 86.50] | <0.001 |
| HbA1c, %, median (IQR) | 5.50 [5.10, 6.60] | 5.30 [5.00, 5.90] | 5.50 [5.10, 6.90] | 5.50 [5.20, 6.20] | 6.10 [5.40, 7.60] | <0.001 |
| FPG, mg/dl, median (IQR) | 140.04 [128.84, 173.70] | 134.28 [126.36, 148.32] | 151.38 [135.63, 203.31] | 128.70 [108.00, 137.34] | 163.26 [140.22, 219.60] | <0.001 |
| TG, mg/dl, median (IQR) | 135.40 [90.27, 220.37] | 84.07 [65.49, 107.97] | 201.78 [150.01, 305.77] | 103.54 [83.63, 123.01] | 229.21 [179.65, 336.30] | <0.001 |
| TC, mg/dl, median (IQR) | 195.62 [169.33, 225.10] | 183.25 [159.28, 212.24] | 201.03 [174.94, 229.83] | 196.01 [167.40, 220.17] | 205.67 [176.29, 238.15] | <0.001 |
| HDL-C, mg/dl, median (IQR) | 44.85 [36.34, 54.51] | 53.35 [43.69, 64.18] | 40.98 [33.44, 50.64] | 47.94 [41.56, 56.06] | 37.11 [30.54, 45.62] | <0.001 |
| LDL-C, mg/dl, median (IQR) | 112.50 [87.76, 139.18] | 112.11 [91.24, 133.38] | 107.47 [76.35, 132.22] | 124.10 [101.48, 147.10] | 105.16 [78.87, 138.79] | <0.001 |
| CRP, mg/l, median (IQR) | 1.22 [0.63, 2.53] | 0.79 [0.44, 1.80] | 1.05 [0.58, 2.29] | 1.37 [0.70, 2.42] | 1.68 [0.95, 3.52] | <0.001 |
| Scr, mg/dl, median (IQR) | 0.76 [0.64, 0.88] | 0.77 [0.63, 0.89] | 0.76 [0.66, 0.89] | 0.76 [0.66, 0.88] | 0.76 [0.66, 0.86] | 0.651 |
| BUN, mg/dl, median (IQR) | 15.27 [12.77, 18.35] | 15.04 [12.41, 18.71] | 15.27 [13.09, 18.33] | 15.46 [13.02, 18.89] | 15.18 [12.94, 17.28] | 0.821 |
| UA, mg/dL, median (IQR) | 4.31 [3.57, 5.20] | 4.23 [3.46, 4.96] | 4.31 [3.65, 5.27] | 4.24 [3.55, 5.23] | 4.42 [3.64, 5.39] | 0.231 |
| TyG, median (IQR) | 9.19 [8.71, 9.83] | 8.65 [8.38, 8.97] | 9.74 [9.47, 10.14] | 8.78 [8.54, 9.00] | 9.87 [9.58, 10.32] | <0.001 |
| BRI, median (IQR) | 4.48 [3.55, 5.55] | 3.43 [2.81, 3.94] | 3.69 [3.16, 4.12] | 5.59 [4.90, 6.16] | 5.53 [5.03, 6.26] | <0.001 |

**Values are mean (SD), median (IQR), or n (%)**

**Abbreviations: BMI: body mass index; SBP: systolic blood pressure; DBP: diastolic blood pressure; HbA1c: glycated hemoglobin; FPG: fasting plasma glucose; TG: triglycerides; TC: total cholesterol; HDL-C: high-density lipoprotein cholesterol; LDL-C: low-density lipoprotein cholesterol; CRP: C-reactive protein; Scr: serum creatinine; BUN: blood urea nitrogen;** UA: uric acid; TyG: **triglyceride glucose; BRI: body roundness index; IQR: interquartile range; SD: standard deviation**

**Table S3** Association of TyG and BRI and the risk of CVD incidence after excluding participants with missing values

| **COX** | **Crude model** | | **Model 1** | | **Model 2** | | **Model 3** | |
| --- | --- | --- | --- | --- | --- | --- | --- | --- |
|  | **HR (95% CI)** | **P value** | **HR (95% CI)** | **P value** | **HR (95% CI)** | **P value** | **HR (95% CI)** | **P value** |
| TyG |  | |  | |  | |  | |
| Low TyG | Ref. |  | Ref. |  | Ref. |  | Ref. |  |
| High TyG | 1.39 (1.04-1.86) | 0.021 | 1.36 (1.01-1.83) | 0.040 | 1.34 (0.99-1.80) | 0.055 | 1.36 (0.98-1.89) | 0.063 |
| BRI |  |  |  |  |  |  |  |  |
| Low BRI | Ref. |  | Ref. |  | Ref. |  | Ref. |  |
| High BRI | 1.91 (1.41-2.59) | <0.001 | 1.79 (1.31-2.46) | <0.001 | 1.76 (1.28-2.41) | <0.001 | 1.83 (1.33-2.51) | <0.001 |
| TyG and BRI |  |  |  |  |  |  |  |  |
| Low TyG & low BRI | Ref. |  | Ref. |  | Ref. |  | Ref. |  |
| High TyG & low BRI | 1.41 (0.87-2.29) | 0.162 | 1.39 (0.86-2.26) | 0.179 | 1.31 (0.81-2.14) | 0.275 | 1.35 (0.82-2.23) | 0.240 |
| Low TyG & high BRI | 2.10 (1.34-3.28) | 0.001 | 1.95 (1.24-3.08) | 0.004 | 1.86 (1.18-2.94) | 0.008 | 1.98 (1.25-3.15) | 0.004 |
| High TyG & high BRI | 2.41 (1.47-3.34) | <0.001 | 2.08 (1.37-3.15) | <0.001 | 2.01 (1.32-3.06) | 0.001 | 2.12 (1.35-3.31) | <0.001 |

Crude model: we did not adjust other covariates

Model 1:we adjusted for age and sex

Model 2: we adjusted for age, sex, smoking status, drinking status, marital status, education level, and residence

Model 3: we adjusted for age, sex, smoking status, drinking status, marital status, education level, residence, CRP, LDL-C, TC, UA, Scr.

**Abbreviations:** TyG: **triglyceride glucose; BRI: body roundness index; CRP: C-reactive protein; LDL-C: low-density lipoprotein cholesterol; TC: total cholesterol;** UA: uric acid; **Scr: serum creatinine; HR: hazard ratio; Ref: reference; CI:** confidence interval**Table S4** Subgroup analysis of the correlation between TyG index and BRI on CVD risk after excluding participants with missing values

|  | Low TyG & low BRI | High TyG & low BRI | Low TyG & high BRI | High TyG & high BRI | P value |
| --- | --- | --- | --- | --- | --- |
| Age |  |  |  |  | 0.199 |
| <60 | Ref | 1.11 (0.53-2.34) | 1.86 (0.93-3.74) | 1.86 (0.96-3.59) |  |
| ≥60 | Ref | 1.37 (0.69-2.71) | 0.97 (0.49-1.94) | 1.04 (0.54-2.01) |  |
| Sex |  |  |  |  | 0.346 |
| Male | Ref | 0.81 (0.39-1.68) | 1.14 (0.52-2.51) | 1.10 (0.53-2.28) |  |
| Female | Ref | 1.63 (0.79-3.34) | 1.49 (0.76-2.93) | 1.45 (0.75-2.80) |  |
| Drinking status |  |  |  |  | 0.144 |
| Current Drinker | Ref | 1.07 (0.44-2.61) | 1.89 (0.73-4.91) | 1.38 (0.55-3.41) |  |
| Former Drinker | Ref | 0.99 (0.20-4.89) | 0.66 (0.12-3.58) | 1.24 (0.28-5.55) |  |
| Never Drinker | Ref | 1.25 (0.63-2.47) | 1.41 (0.76-2.63) | 1.38 (0.75-2.52) |  |
| Smoking status |  |  |  |  | 0.116 |
| Current Smoker | Ref | 0.88 (0.34-2.27) | 1.80 (0.68-4.75) | 2.00 (0.82-4.85) |  |
| Former Smoker | Ref | 0.10 (0.01-1.48) | 3.38 (0.74-15.37) | 0.39 (0.07-2.22) |  |
| Never Smoked | Ref | 1.93 (0.98-3.79) | 1.38 (0.72-2.66) | 1.49 (0.80-2.78) |  |
| Dyslipidemia |  |  |  |  | 0.012 |
| No | Ref | 1.41 (0.83-2.40) | 1.60 (0.94-2.75) | 1.50 (0.89-2.52) |  |
| Yes | Ref | 0.30 (0.06-1.36) | 0.34 (0.09-1.23) | 0.53 (0.17-1.63) |  |
| Hypertension |  |  |  |  |  |
| No | Ref | 1.33 (0.72-2.46) | 1.47 (0.77-2.82) | 1.50 (0.84-2.68) | 0.514 |
| Yes | Ref | 1.06 (0.44-2.56) | 1.25 (0.56-2.80) | 1.23 (0.56-2.72) |  |

**Abbreviations:** TyG:**triglyceride glucose; BRI: body roundness index; Ref: referenceTable S5** Baseline characteristics grouped by cumulative TyG index and cumulative BRI

| **Characteristics** | **All** | **Low TyG & low BRI** | **High TyG & low BRI** | **Low TyG & high BRI** | **High TyG & high BRI** | **P value** |
| --- | --- | --- | --- | --- | --- | --- |
| n | 743 | 229 | 142 | 142 | 230 |  |
| Age,yearss, median (IQR) | 59.00 [54.00, 64.00] | 59.00 [54.00, 66.00] | 58.00 [53.00, 61.00] | 60.00 [55.25, 68.00] | 59.00 [53.00, 64.00] | 0.002 |
| Sex (Female), n (%) | 402 (54.1) | 87 (38.0) | 63 (44.4) | 98 (69.0) | 154 (67.0) | <0.001 |
| Current married, n (%) | 639 (86.0) | 193 (84.3) | 120 (84.5) | 121 (85.2) | 205 (89.1) | 0.428 |
| Education level, n (%) |  |  |  |  |  | 0.271 |
| Elementary school or below | 531 (71.5) | 163 (71.2) | 91 (64.1) | 102 (71.8) | 175 (76.1) |  |
| Middle school | 193 (26.0) | 60 (26.2) | 48 (33.8) | 35 (24.6) | 50 (21.7) |  |
| College or above | 19 (2.6) | 6 (2.6) | 3 (2.1) | 5 (3.5) | 5 (2.2) |  |
| Residence, n (%) |  |  |  |  |  | 0.247 |
| Urban | 111 (14.9) | 27 (11.8) | 24 (16.9) | 27 (19.0) | 33 (14.3) |  |
| Rural | 632 (85.1) | 202 (88.2) | 118 (83.1) | 115 (81.0) | 197 (85.7) |  |
| Smoking status, n (%) |  |  |  |  |  | <0.001 |
| Current Smoker | 211 (28.5) | 97 (42.4) | 45 (31.9) | 26 (18.4) | 43 (18.8) |  |
| Former Smoker | 67 (9.1) | 18 (7.9) | 15 (10.6) | 12 (8.5) | 22 (9.6) |  |
| Never Smoked | 462 (62.4) | 114 (49.8) | 81 (57.4) | 103 (73.0) | 164 (71.6) |  |
| Drinking status, n (%) |  |  |  |  |  | <0.001 |
| Current Drinker | 239 (32.2) | 89 (39.0) | 63 (44.4) | 32 (22.5) | 55 (23.9) |  |
| Former Drinker | 71 (9.6) | 26 (11.4) | 10 (7.0) | 13 (9.2) | 22 (9.6) |  |
| Never Drinker | 432 (58.2) | 113 (49.6) | 69 (48.6) | 97 (68.3) | 153 (66.5) |  |
| Psych problem, n (%) | 6 (0.8) | 1 (0.4) | 1 (0.7) | 1 (0.7) | 3 (1.3) | 0.939 |
| Dyslipidemia, n (%) | 111 (15.1) | 15 (6.6) | 13 (9.3) | 16 (11.6) | 67 (29.4) | <0.001 |
| Hypertension, n (%) | 254 (34.3) | 48 (21.0) | 35 (25.0) | 65 (45.8) | 106 (46.3) | <0.001 |
| Kidney disease, n (%) | 33 (4.5) | 9 (3.9) | 10 (7.1) | 7 (4.9) | 7 (3.0) | 0.296 |
| Liver disease, n (%) | 22 (3.0) | 8 (3.5) | 1 (0.7) | 4 (2.8) | 9 (3.9) | 0.301 |
| Lunge disease, n (%) | 58 (7.8) | 24 (10.5) | 7 (5.0) | 9 (6.4) | 18 (7.8) | 0.261 |
| Digestive disease, n (%) | 148 (19.9) | 46 (20.1) | 29 (20.6) | 27 (19.0) | 46 (20.0) | 0.990 |
| Cancer, n (%) | 8 (1.1) | 1 (0.4) | 2 (1.4) | 0 (0.0) | 5 (2.2) | 0.203 |
| Dyslipidemia medications, n (%) | 61 (8.3) | 7 (3.1) | 9 (6.4) | 10 (7.3) | 35 (15.4) | <0.001 |
| Hypertension medications, n (%) | 192 (25.9) | 28 (12.2) | 27 (19.3) | 51 (35.9) | 86 (37.6) | <0.001 |
| BMI,kg/m^2^, median (IQR) | 24.22 [21.86, 26.86] | 21.53 [20.02, 23.25] | 22.98 [21.30, 24.70] | 26.64 [24.09, 28.55] | 26.67 [24.72, 28.89] | <0.001 |
| SBP, mmHg, median (IQR) | 131.25 [117.88, 145.00] | 128.50 [113.50, 141.12] | 128.75 [116.25, 141.00] | 136.00 [120.00, 146.50] | 134.50 [122.00, 151.50] | <0.001 |
| DBP, mmHg, median (IQR) | 76.50 [68.50, 83.50] | 73.50 [66.50, 80.62] | 74.25 [68.50, 82.88] | 77.00 [71.00, 83.50] | 79.00 [72.00, 86.50] | <0.001 |
| HbA1c, %, median (IQR) | 5.50 [5.10, 6.60] | 5.25 [4.90, 5.70] | 5.90 [5.20, 7.60] | 5.50 [5.20, 6.10] | 5.90 [5.40, 7.30] | <0.001 |
| FPG, mg/dl, median (IQR) | 140.40 [128.79, 175.14] | 134.46 [126.90, 148.50] | 158.22 [136.71, 217.03] | 130.14 [112.45, 142.38] | 158.58 [134.01, 204.52] | <0.001 |
| TG, mg/dl, median (IQR) | 137.18 [91.15, 226.12] | 88.50 [68.14, 121.25] | 218.15 [146.47, 364.18] | 107.08 [84.30, 127.22] | 220.81 [160.41, 335.86] | <0.001 |
| TC, mg/dl, median (IQR) | 195.23 [170.10, 225.77] | 183.25 [160.44, 207.22] | 204.90 [177.84, 235.25] | 191.37 [169.81, 222.20] | 206.64 [177.16, 236.50] | <0.001 |
| HDL-C, mg/dl, median (IQR) | 44.07 [35.57, 54.51] | 52.19 [44.07, 63.79] | 38.66 [31.80, 47.07] | 49.68 [41.37, 56.73] | 36.15 [29.77, 45.52] | <0.001 |
| LDL-C, mg/dl, mean (SD) | 112.11 [88.14, 139.18] | 108.25 [89.69, 131.44] | 107.86 [74.61, 134.92] | 120.43 [101.00, 147.20] | 109.79 [85.63, 139.76] | <0.001 |
| CRP, mg/l, median (IQR) | 1.25 [0.64, 2.54] | 0.79 [0.47, 1.87] | 1.39 [0.67, 2.54] | 1.31 [0.73, 2.23] | 1.53 [0.88, 3.45] | <0.001 |
| Scr, mg/dl, median (IQR) | 0.75 [0.64, 0.87] | 0.77 [0.63, 0.89] | 0.77 [0.67, 0.89] | 0.72 [0.66, 0.86] | 0.73 [0.64, 0.84] | 0.138 |
| BUN, mg/dl, median (IQR) | 15.29 [12.79, 18.46] | 15.35 [12.77, 19.07] | 15.46 [12.98, 18.23] | 15.43 [13.26, 18.83] | 14.96 [12.72, 17.53] | 0.352 |
| UA, mg/dL, median (IQR) | 4.29 [3.58, 5.20] | 4.19 [3.46, 5.04] | 4.39 [3.68, 5.61] | 4.21 [3.54, 5.21] | 4.38 [3.67, 5.18] | 0.179 |
| Cumulative TyG, median (IQR) | 9.20 [8.70, 9.86] | 8.67 [8.42, 9.03] | 9.86 [9.47, 10.41] | 8.82 [8.53, 9.04] | 9.84 [9.48, 10.29] | <0.001 |
| Cumulative BRI, median (IQR) | 4.55 [3.61, 5.56] | 3.49 [2.86, 4.02] | 3.79 [3.28, 4.27] | 5.60 [4.91, 6.15] | 5.52 [4.93, 6.26] | <0.001 |

**Values are mean (SD), median (IQR), or n (%)**

**Abbreviations: BMI: body mass index; SBP: systolic blood pressure; DBP: diastolic blood pressure; HbA1c: glycated hemoglobin; FPG: fasting plasma glucose; TG: triglycerides; TC: total cholesterol; HDL-C: high-density lipoprotein cholesterol; LDL-C: low-density lipoprotein cholesterol; CRP: C-reactive protein; Scr: serum creatinine; BUN: blood urea nitrogen;** UA: uric acid; TyG: **triglyceride glucose; BRI: body roundness index; IQR: interquartile range; SD: standard deviation**

| **Logistics** | **Crude model** | | **Model 1** | | **Model 2** | | **Model 3** | |
| --- | --- | --- | --- | --- | --- | --- | --- | --- |
|  | **OR (95% CI)** | **P value** | **OR (95% CI)** | **P value** | **OR (95% CI)** | **P value** | **OR (95% CI)** | **P value** |
| TyG |  | |  | |  | |  | |
| Low TyG | Ref. |  | Ref. |  | Ref. |  | Ref. |  |
| High TyG | 1.46(1.05-2.05) | 0.026 | 1.45(1.03-2.04) | 0.033 | 1.44(1.02-2.04) | 0.039 | 1.49(1.03-2.16) | 0.034 |
| BRI |  |  |  |  |  |  |  |  |
| Low BRI | Ref. |  | Ref. |  | Ref. |  | Ref. |  |
| High BRI | 1.97(1.41-2.79) | <0.001 | 1.81(1.27-2.59) | 0.001 | 1.75(1.22-2.51) | 0.002 | 1.76(1.22-2.54) | 0.002 |
| TyG and BRI |  |  |  |  |  |  |  |  |
| Low TyG & low BRI | Ref. |  | Ref. |  | Ref. |  | Ref. |  |
| High TyG & low BRI | 1.51(0.89-2.56) | 0.126 | 1.51(0.88-2.57) | 0.130 | 1.34(0.89-2.02) | 0.130 | 1.46(0.85-2.51) | 0.167 |
| Low TyG & high BRI | 2.11(1.27-3.50) | 0.004 | 1.90(1.33-3.20) | 0.015 | 1.78(1.21-2.62) | 0.015 | 1.77(1.05-3.00) | 0.033 |
| High TyG & high BRI | 2.46(1.58-3.88) | <0.001 | 2.29(1.45-3.65) | <0.001 | 2.04(1.43-2.91) | <0.001 | 2.21(1.39-3.54) | <0.001 |

**Table S6** The correlation between cumulative TyG index and cumulative BRI with CVD incidence risk based on logistics regression model

Crude model: we did not adjust other covariates

Model 1:we adjusted for age and sex

Model 2: we adjusted for age, sex, smoking status, drinking status, marital status, education level, and residence

Model 3: we adjusted for age, sex, smoking status, drinking status, marital status, education level, residence, CRP, LDL-C, TC, UA, Scr.

**Abbreviations:** TyG: **triglyceride glucose; BRI: body roundness index; CRP: C-reactive protein; LDL-C: low-density lipoprotein cholesterol; TC: total cholesterol;** UA: uric acid; **Scr: serum creatinine; OR: odds ratio; Ref: reference; CI: confidence interval**

**Table S7** Subgroup analysis of the correlation between cumulative TyG index and cumulative BRI on CVD risk

|  | Low TyG & low BRI | High TyG & low BRI | Low TyG & high BRI | High TyG & high BRI | P value |
| --- | --- | --- | --- | --- | --- |
| Age |  |  |  |  | 0.105 |
| <60 | Ref | 1.68 (0.73-3.96) | 2.23 (0.93-5.42) | 1.61 (0.74-3.57) |  |
| ≥60 | Ref | 1.12 (0.48-2.52) | 0.93 (0.43-1.98) | 1.34 (0.66-2.76) |  |
| Sex |  |  |  |  | 0.607 |
| Male | Ref | 1.14 (0.48-2.63) | 1.59 (0.62-3.98) | 1.38 (0.58-3.21) |  |
| Female | Ref | 1.78 (0.77-4.12) | 1.10 (0.51-2.38) | 1.34 (0.66-2.76) |  |
| Drinking status |  |  |  |  | 0.034 |
| Current Drinker | Ref | 0.00 (0.00-0.00) | 0.00 (0.00-0.00) | 0.00 (0.00-0.00) |  |
| Former Drinker | Ref | 1.51 (0.16-12.09) | 0.23 (0.02-2.15) | 1.13 (0.19-6.36) |  |
| Never Drinker | Ref | 1.00 (0.44-2.25) | 1.21 (0.59-2.49) | 1.29 (0.66-2.55) |  |
| Smoking status |  |  |  |  | 0.220 |
| Current Smoker | Ref | 1.26 (0.41-3.73) | 1.09 (0.30-3.60) | 2.46 (0.87-7.04) |  |
| Former Smoker | Ref | 0.00 (0.00-0.00) | 0.00 (0.00-0.00) | 0.00 (0.00-0.00) |  |
| Never Smoked | Ref | 1.83 (0.86-3.96) | 1.21 (0.59-2.52) | 1.36 (0.70-2.71) |  |
| Dyslipidemia |  |  |  |  | 0.045 |
| No | Ref | 1.65 (0.89-3.08) | 1.52 (0.82-2.82) | 1.73 (0.97-3.13) |  |
| Yes | Ref | 0.24 (0.03-1.51) | 0.17 (0.03-1.00) | 0.30 (0.07-1.22) |  |
| Hypertension |  |  |  |  |  |
| No | Ref | 1.61 (0.77-3.34) | 1.39 (0.61-3.06) | 1.47 (0.73-3.00) | 0.654 |
| Yes | Ref | 1.20 (0.45-3.19) | 1.22 (0.52-2.90) | 1.43 (0.64-3.29) |  |

**Abbreviations:** TyG:**triglyceride glucose; BRI: body roundness index; Ref: reference**
